# Correlations of thought disorders with attenuated positive symptoms at clinical high-risk state for psychosis

**DOI:** 10.1192/j.eurpsy.2023.627

**Published:** 2023-07-19

**Authors:** V. Kaleda, M. Omelchenko, E. Arutyunova

**Affiliations:** ^1^Department of youth psychiatry, Federal State Budgetary Scientific Institution Mental Health Research Center, Moscow, Russian Federation

## Abstract

**Introduction:**

One of the methods for early diagnosis in individuals at clinical high-risk state for psychosis could be the identifying specific symptoms, such as thought disorders. Thought disorders may be a separate symptom, unrelated to attenuated positive symptoms (APS) with an independent predictive value.

**Objectives:**

Correlation analysis of thought disorders and APS in patients at clinical high-risk state for psychosis.

**Methods:**

The study included 30 young men (mean age 19.2±2.1 years) hospitalized at the clinic of the FSBSI “Mental Health Research Centre” with the APS in the first depressive episode (F32.1, F32.2, F32.28, F32.8) which is considered as clinical high-risk state for psychosis. The severity of thought impairment was assessed using the Thought, Language and Communication Scale (TLC). Subsequently was performed the search for correlations of scores on the TLC and the severity of “prodromal” symptoms, according to The Scale of Prodromal Symptoms (SOPS).

**Results:**

The median value of the total score on the TLC was 20 [17.25;23.5]. The most important finding is the discovery of only minor correlations of thought disorder with “prodromal” symptoms. Indeed, the total score on the TLC correlated only with the total score on the SOPS at admission (r=0.370, p<0.05). Such symptoms of the TLC scale as «Derailment», «Incoherence», «Perseveration» did not find any correlation with prodromal symptoms (p>0.05).

**Conclusions:**

The obtained data indicates the independent nature of thought disorder in patients at clinical high-risk state for schizophrenia, which leads the need to determine its own prognostic value.

**Disclosure of Interest:**

None Declared

